# First description of behavior and immune system relationship in fish

**DOI:** 10.1038/s41598-018-19276-3

**Published:** 2018-01-16

**Authors:** Karina Kirsten, Débora Fior, Luiz Carlos Kreutz, Leonardo José Gil Barcellos

**Affiliations:** 1 Programa de Pós-Graduação em Farmacologia, Universidade Federal de Santa Maria (UFSM), Av. Roraima, 1000, Cidade Universitária, Camobi, Santa Maria, RS 97105-900 Brazil; 2 Laboratório de Microbiologia e Imunologia Avançada, Programa de Pós-Graduação em Bioexperimentação, Universidade de Passo Fundo (UPF), BR 285, São José, Passo Fundo, RS 99052-900 Brazil; 3 Programa de Pós-Graduação em Ciências Ambientais and Programa de Pós-Graduação em Bioexperimentação, Universidade de Passo Fundo, (UPF), BR 285, São José, Passo Fundo, RS 99052-900 Brazil

## Abstract

Considering the intriguing relationship between immune system and behavior recently described in mammals, and the lack of information of this relationship in fish, here we describe for the first time the interaction between the immune system and social and exploratory behavior in zebrafish. Fish high responders to novelty (HRN) presented a proinflammatory profile, with increased IL-1β and reduced IL-10 expression compared to fish low responders to novelty (LRN). Likewise, fish less responsive to social stimuli have a reduced expression of INF-γ. We show that fish with different behavior patterns have differences in the immune response. Our findings indicate that the interplay between immune system and behavior in zebrafish is similar to that found in mammalian models and that zebrafish should be considered as a potential model organism to study the relationship between immune system and behavior.

## Introduction

The immune system and behavior are deeply connected. Human behavioral disorders such as autism, schizophrenia and depression are associated with dysfunction of the immune system, with altered levels of cytokines, which in turn might directly affect neuronal function^1–3^. While the capacity of neurological diseases to interfere with immune system is widely studied^4^ but, in contrast, investigations on the role of the immune system in regulating neuronal physiology are only emerging recently^5,6^. Studies in rodents demonstranted that cells and molecules of the immune system are involved in neurological functions such as learning, memory, anxiety and social behavior^5–9^. Mast cells, which are involved in allergic responses, are present in meninges and brain and are key cells during neurogenesis: a deficiency in mast cells increases anxiety, and causes learning and memory deficits^7^. Learning and memory are also affected by Interleukin 4 (IL-4), a cytokine produced by mast cells during inflammation, and by T lymphocytes, which are found on meninges and central to adaptive immune responses^8^. Furthermore, Interferon gamma (INF-γ), a pleiotropic cytokine also produced by T lymphocytes is involved in controlling social behavior^9^.

Although the interaction of nervous and immune system has been investigated using mammals as animal models, there is a potential to exploit other species as model organisms. The zebrafish is one of the main animals species used in scientific research^10^, widely employed in behavioral studies^11^, because it presents behavior characteristics of other vertebrates^12^, clear individual differences of behavior^13^, besides there are several behavioral tests standardized for scientific use in the species^14^. Considering the intriguing relationship between immune system and behavior^3,5–9,15^, and the lack of information of this relationship in fish, here we describe the interaction between the immune system and social and exploratory behavior in zebrafish.

## Results

### Novel object test - immune system x exploratory behavior

**Figure 1:**
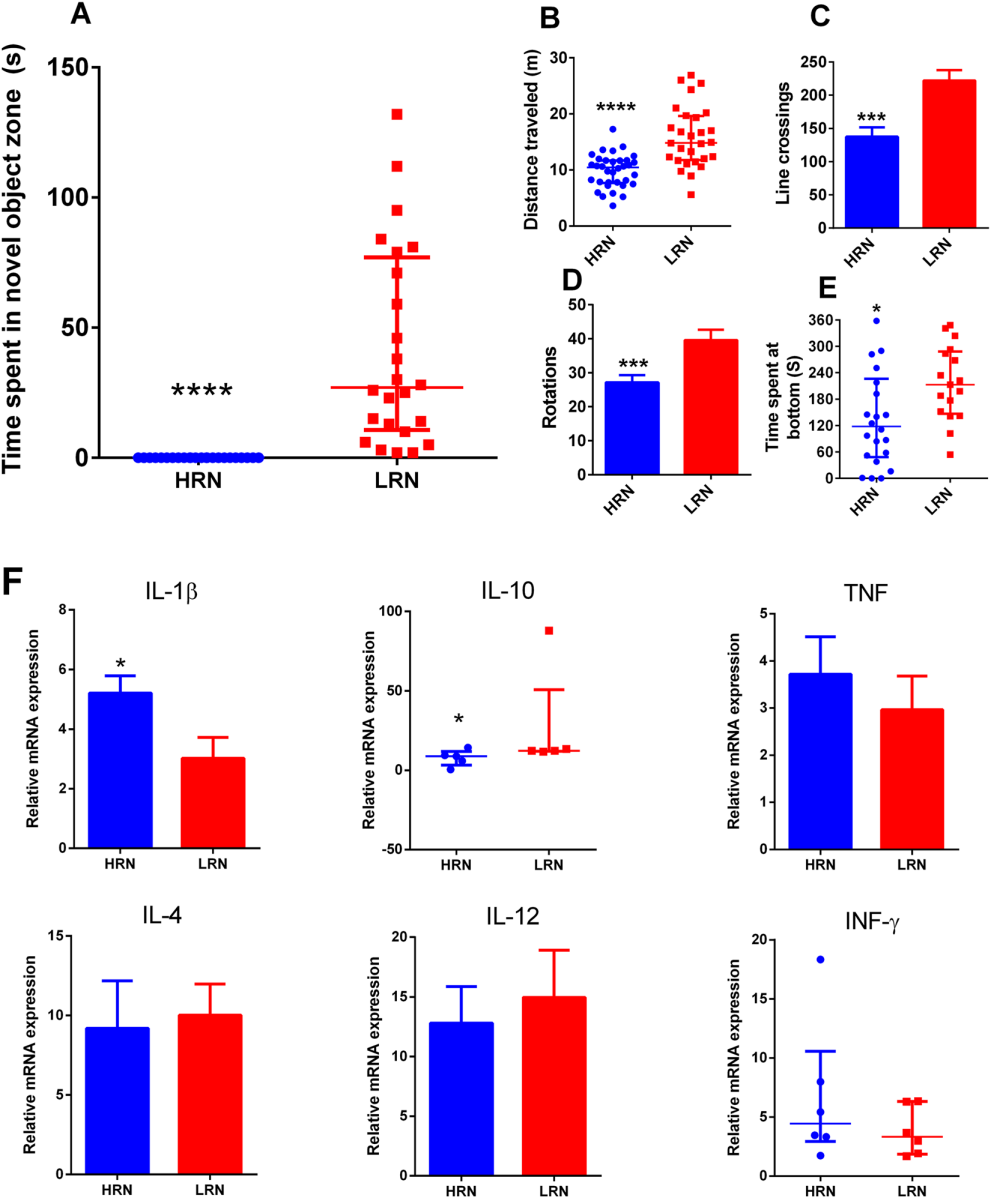
Novel object test. Time spent in the novel object zone (**A**), distance traveled (**B**), line crossings (**C**), rotations (**D**), time spent at the bottom (**E**) and expression of cytokine genes (**F**). Each data represents the mean ± SEM or median ± interquartile range, depending on the data normality assessed by the Bartlett’s test. In panels A to E data represents the mean or median of 24 fish. In panel F, data represents the mean or median of 6 pooled samples. Significant differences are indicated by asterisk (*p < 0.05; ****p < 0.0001).

The time spent in the presence of a novel object was significantly lower in fish classified as high responders to novelty (HRN) (Wald-Wolfowitz rank test, P < 0.0001, Fig. 1A). These HRN fish also presented differences in locomotor parameters as decreased distance traveled (1B), line crossings (1C) and rotations (1D), and spent less time at the tank bottom (1E). In the HRN fish, the mRNA levels of IL-1β were upregulated while the levels of IL-10 mRNA were downregulated indicating a proinflammatory profile. No significant differences were found in the mRNA levels of the other cytokines tested (1F). The raw data and statistics are presented as Supplementary Information.

### Social preference test - immune system x social behavior

**Figure 2:**
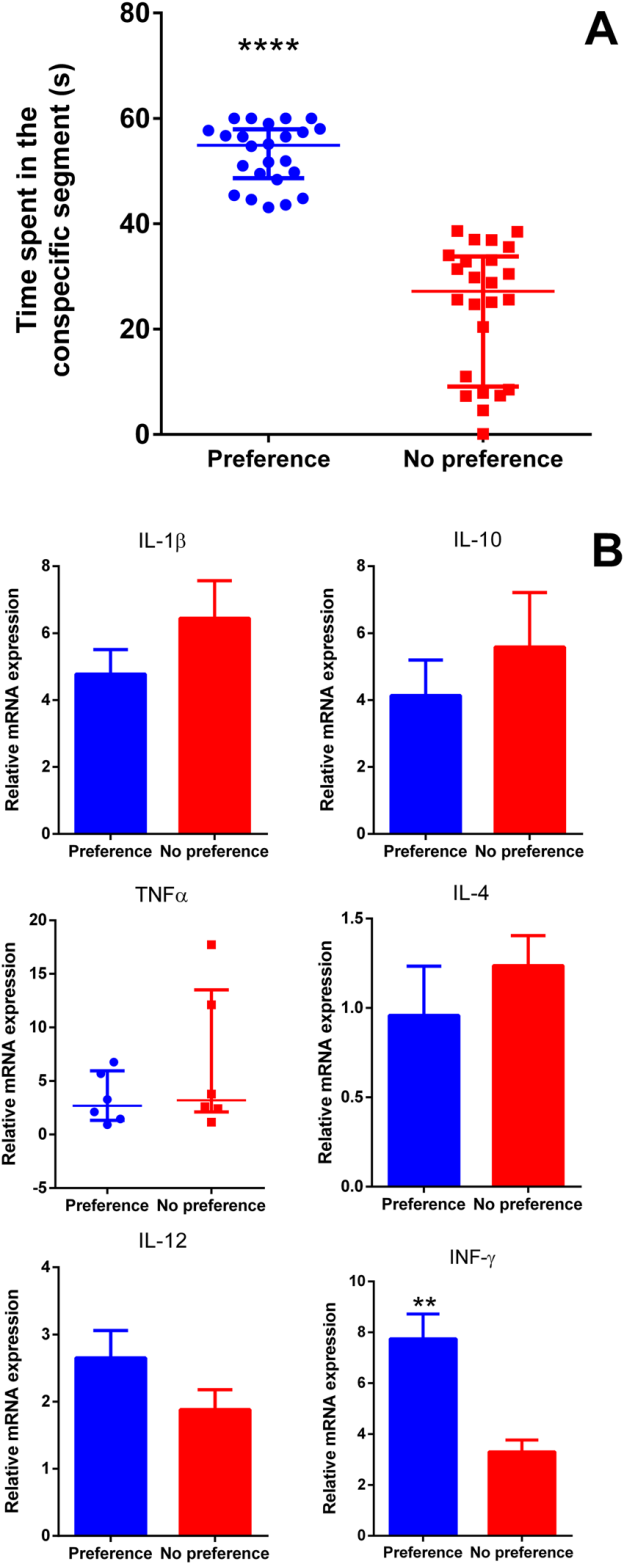
Social preference test- time spent in the conspecific segment. (**A**), and gene expression of cytokines (**B**). Each data represents the mean ± SEM or median ± interquartile range, depending on the data normality assessed by the Bartlett’s test. In panel A data represents the median of 24 fish and in panel B data represents the mean or median of 6 pooled samples. Significant differences are represented by asterisk (**p < 0.01; ****p < 0.0001).

Zebrafish exhibiting a “no preference” profile spent less time in the segment close to their conspecifics (p < 0.0001) (Fig. 2A). In these fish without social preference, the INF-γ gene expression was significantly reduced (p < 0.01). No differences in the mRNA levels were detected in the other genes evaluated (Fig. 2B). The raw data and statistics are provided as Supplementary Information.

## Discussion

Here we show a clear relationship between the immune response of zebrafish and their exploratory and social behavior. Fish high responders to novelty (HRN) express cytokines with a proinflammatory profile, with increased IL-1β and reduced IL-10 expression compared to fish low responders to novelty (LRN). Likewise, fish less responsive to social stimuli have a reduced expression of INF-γ. Despite the clear relationship between the expression of selective immune-related genes and behavior, an intriguing question arises: is the immune system that leads to different behavioral patterns or is the behavioral pattern that alters cytokine profile and the outcome of the immune response?

Our main hypothesis is that the immune system modulates fish behavior. Challenging pathogens trigger a complex set of innate and adaptive immune mechanisms interconnected by cytokines secreted by several immune cells^16^. Besides promoting resistance to pathogens these cytokines modulate behavior to minimize the risk of new infections^4,17^. This cytokine-driven “sickness behavior”, that is a set of behavioral changes described in mammals, characterized by reduces social interaction, water and food intake and exploration of new environments and new objects^2,18^. This proinflammatory profile supports previous work suggesting that neophobia (HRN fish) may be a behavioural defense mechanism, lowering both risk of predation and exposure to infection through reduced exposure^19–21^.

HRN fish presented lower locomotor activity and remained less time at the bottom of the tank compared to LRN fish. At a simplistic view, LRN fish seems to exhibit an anxiety-like behavior^14^. However, we hypothesized that the increased locomotor activity observed with the LRN fish reflects their response to the new object. As they are exploring, they spend more time at the bottom of the tank where the new object is located. On the other hand, HRN fish may be avoiding the new object segment moving less and staying longer in the top of the tank, keeping a greater distance from the new object.

In the social behavior test, fish that spent less time close to their conspecific, had reduced levels of INF-γ mRNA. Interestingly, a recent study demonstrated that INF-γ knockout mice displayed reduced social behavior, indicating the central role of this cytokine on social interaction^6^. That study also demonstrated a relationship between the expression of IFN- γ and the social context in zebrafish^6^. Although social behaviour is crucial for survival and reproduction in many species^19^, it is also well documented that increasing exposure to conspecifics increases the spread of pathogens^6^.

Thus, it would be expected that increased social interactions would be indicative of a robust expression of immune related genes, mainly INF-γ, which appears as a key molecule in this interplay. Nonetheless, although we demonstrated the immune-behavioral relationship, we cannot ascertain weather the reduction of INF-γ leads the fish to respond less to social stimuli, or its lower interaction with the school reflects lower expression of INF-γ. Likewise, HRN behavior in fish results from the expression of proinflammatory cytokine genes or it is their fearful, stressing-type of behavior that leads to the expression IL-1B? There are evidences supporting both theories. Behavioral changes were observed in animals knockout for specific immune-related genes: for instance, lack of IL-4 expression might cause learning and memory deficits, and anxiolytic disorders^5,8^; IL-1β receptor knockouts have memory deficits^9^, and social dysfunction was observed in mice knockout for INF-γ^6^. Thus, at infection, the sudden rise on cytokine expression needed to mount an immune response to overcome threatening pathogens leads to behavioral changes known as sickness behavior^17^. These data support the hypothesis that cytokines, mastering communication between cells and tissues, act also upon the nervous system causing behavioral changes.

On the other hand, behavioral changes may also alter the immune response. It has been shown that social status (dominant vs. subordinate) leads to different patterns of immune response^20,21^. Subordinated animals have a profile of proinflammatory immune cells compared to their dominant cohorts. Furthermore, when the social hierarchy is changed, the immune system alters equally, so it appears that behaviour can also modulate immune response in some situations^20^. Thus, it is likely that subordinated animals are more stressed and, consequently, prone to have increased inflammatory response. Furthermore, several studies in humans showed an imbalance of cytokines concomitantly with pathologies such as depression and autism^3,15,22–24^.

Thus, neurological disturbances might alter functioning of the immune system, and immune dysfunctions can lead to behavioral changes. There is not a relationship of cause-effect, but an intimate interaction between the immune and nervous systems and little is known about the molecular pathways involved in this relationship.

Here, the relationship between behavior and immune system is described for the first time in fish. We show that the expression of specific cytokine genes in the brain of fishes varies according to behavior pattern. Our findings show that neuro-immunological interplay in fish is similar to current mammalian models highlighting the potential of zebrafish as model organism to study immune-behavioral relationship. In an ecological perspective, fish with an immune suppression cytokine profile would reduce interaction with conspecifics in several aspects of their social behavior like mating^25^, hierarchical contest^26^ and defense shoaling^27^. Immune suppressed fish might also have difficulties in tuning risk assessment of the environment during their exploratory activity in search for food and, as a consequence, become more susceptible to predators^28^. Aquatic contaminants like agrichemicals and drugs are known to alter the expression of specific cytokine genes^29–32^ which, in addition to increasing susceptibility to infections, might cause behavioral changes, letting the fish more vulnerable to predation. In this scenario, immune dysfunctions when associated with behavioral changes would have a greater ecological impact and could contribute to reduce fish population in the wilderness.

## Materials and Methods

### Study strategy

To evaluate the relationship between behavior and immune systems, we firstly discriminated zebrafish according to their exploratory behavior (high and low responders to novelty, HRN and LRN respectively) and according to their social behavior (preference and no preference for conspecifics). After, we evaluated the gene expression of selected cytokines in the brain to verify if there were differences in the immunological status in fish presenting different behaviors.

### Animals and Maintenance

A stock population of 195 180-day old adult wild-type zebrafish (*Danio rerio*), mixed sex (50:50) from a single brood, were held in a tank equipped with biological filters, with a natural photoperiod (14 h light:10 h dark) and under constant aeration. The fish were fed twice a day with commercial flaked food provided *ad libidum*. Water temperature was maintained at 26 ± 2 C°, dissolved oxygen concentrations at 6.5 ± 0.4 mg/L, pH 7.0 ± 0.25, and the total ammonia concentration was less than 0.5 mg/L. Fish used in the study were in perfect health and were not subjected to any procedure or exposure to drugs prior to the experiments.

### Behavioral evaluation

Two behavioral tests were performed in different zebrafish groups: the new object test, to classify fish into HRN and LRN in relationship to their exploratory behavior, and the social preference test, to discriminate fish according to their response to social stimuli. In both tests, fish behavior was recorded by a Logitech Quickcam PRO 9000 camera located in front of the tank, and the videos analyzed using ANY-maze® software (Stoelting CO, USA), which tracked animal behavior throughout testing. After testing, the water of the tank was completely changed prior to testing a new fish.

### Novel object test

**Figure 3:**
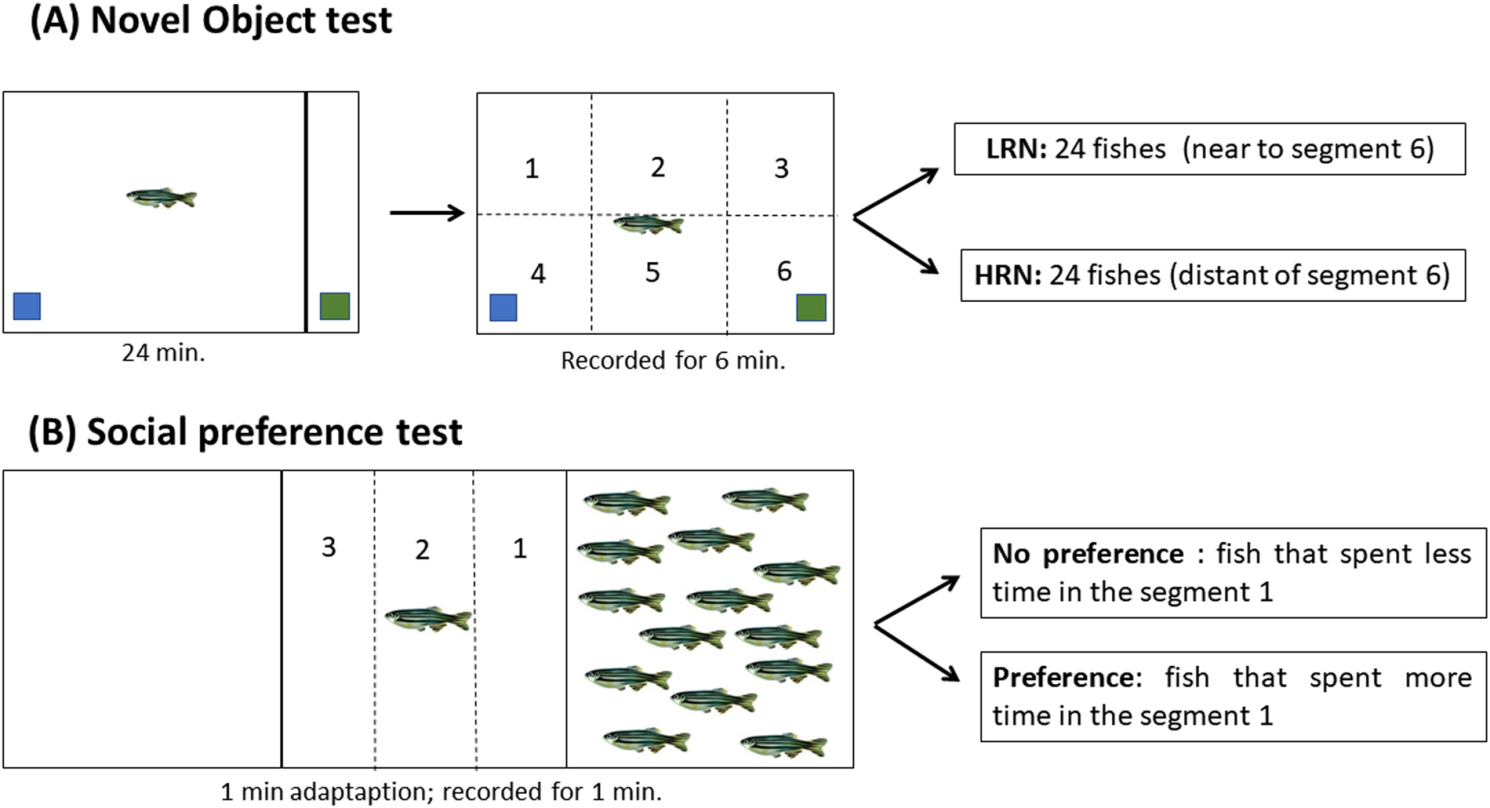
Schematic representation of the methodology used to discriminate exploratory (**A**) and social (**B**) behavior of zebrafish. The drawings in the panels A and B were drawn by KK.

A total of 120 fish were tested to determine their responsiveness to novelty. The protocol was adapted from Braida *et al*.^33^ and May *et al*.^34^. Each fish was individually transferred to the test tank (24 × 8 × 20 cm; width × depth × height), which contained one object at each extremity (blue and green plastic squares measuring 4 × 4 cm). One of the object was covered by an opaque partition close to the extremity and could not be seen by the fish. The fish was introduced into the tank and for 24 minutes (based in the time to memory acquisition^35^) it was in contact with only one object. Then, the partition was removed and fish had access to the “new object”. After partition removal, the behavior was recorded for 6 minutes. The color of the new object (blue or green) varied in each test. For the analysis, the test tank was virtually divided into six segments and the fish were classified by the time spent in the segment of the new object. We considered as HRN the 24 fish that did not enter once in the segment of the new object and as LRN the 24 fish that stayed longer next to the new object (Fig. 3A). We analyzed also the total distance traveled (m), number of line crossings, number of rotations and time in the bottom part of the tank (s).

### Social preference test

A total of 60 fish were tested to determine their preference for conspecifics. In this test, fish were transferred individually to the test tank (30 × 15 × 10 cm; width × depth × height). The test tank was positioned between two equal sized tanks, one without fish and the other containing 15 conspecifics^36^. After transferring to the test tank, fish were acclimated for 60 seconds and their behavior was recorded for 60 seconds. For the image analysis, the test tank was virtually divided into three vertical segments. The first segment was the one nearest to conspecifics, while the third segment was next to the empty tank. The relative time zebrafish spent in the first segment was calculated as response to social stimuli (Fig. 3B).

### Euthanasia and brain extraction

Immediately after performing the behavioral tests, each fish was anesthetized with Eugenol (Sigma Aldrich, Brazil, 50 mg/L) and euthanized by sectioning the spinal cord. Then, each fish was separately packed in microtubes, frozen in liquid nitrogen for 30 seconds and stored at −80 °C. For brain extraction, the skull was opened, the cranial nerves severed, and the brain carefully removed^37^.

### RNA extraction and cDNA synthesis

Due to the small size, the brains of four fish with the same behavioral profile were randomly pooled and used for total RNA extraction, totaling six RNA samples per group (24 fish) (6 samples per group). Tissue lysis was done using the Tissuelyser LT® (Qiagen, Brazil). RNA was extracted using RNeasy® Mini Kit (Qiagen, Brazil) and submitted to a DNAse I amplification grade treatment (Invitrogen, EUA) to eliminate genomic DNA. The RNA quality and concentration was measured by spectrophotometry (Nanophotometer Pearl®, IMPLEN, Germany), and stored at −80 °C. For cDNA synthesis, 500 ng of total RNA was used for the reverse transcription assay, using SuperScript® III Reverse Transcriptase (Invitrogen, EUA) and random primers.

### Gene expression analysis by real-time quantitative PCR (qPCR)

Real time PCR (qPCR) was performed in 48-well plates (MicroAmp Fast optical 48 well plate reaction, 0.1 ml. Applied Biosystems, USA) in a final volume of 10 µl. The mix consisted of 400 nM of each primer, 5 µl of SYBR Select Master Mix (Applied Biosystems, USA) and 1 µl of cDNA (diluted 1:10). The reaction was carried out in a Step One equipment Applied Biosystems) with the following conditions: initial denaturing at 95 °C for 10 min followed by 40 cycles of 95 °C for 30 s, 60 °C for 30 s, and 72 °C for 30 s. At the end, a standard melting curve was included to confirm the specificity of the amplified product. Samples were performed in triplicate. The genes analyzed, their respective primers and the efficiency of each reaction are indicated in Table 1. Non template controls and the expression of a housekeeping gene (β-actin) were also analysed for comparison purposes. For the calibration curve, each gene was cloned into the pGEM-TEasy Vector System (Promega) and transformed into competent One Shot TOP10 E. coli (Promega) and cultured in LB supplemented with ampicillin. Cloning was confirmed by PCR and the resulting plasmid was extracted using the Wizard Plus SV minipreps DNA purification system (Promega). Then, the calibration curve consisted of decimal dilutions (1:10) of each cloned gene. To better compare the results from different groups the same threshold value (0.080) was used. The relative quantification of gene expression, was carried out by the 2^−ΔΔct^ formula^38^.

**Table 1:** Immune genes, primers nucleotide sequence and qPCR efficiency.

Gene	Primer (5′-3′)	Efficiency	Accession number
TNF-α	F: GACCACAGCACTTCTACCG R: ACATTTTCCTCACTTTCGTTCAC	98,3%	NM_212859
IL-1β	F: GCTGGAGATGTGGACTTC R: ACTCTGTGGATTGGGGTTTG	102%	NM_212844
INF-γ	F: TGCCTCAAAATGGTGCTACTC R: AATCGGGTTCTCGCTCCTG	97,1%	AB158361.1
IL-4	F: TCTCTGCCAAGCAGGAATG R: CAGTTTCCAGTCCCGGTATATG	99,4%	AM403245.2
IL-12	F: CTGTAGGATCCATCCAAACATCT R: CACTGGCACTTCTACCCTATTT	96,8%	AB183002.1
IL-10	F: CTCTGCTCACGCTTCTTCTT R: GCTCCCTCAGTCTTAAAGGAAA	101,4%	BC163038.1
β-Actin	F: GCAAAGGGAGGTAGTTGTCTAA R: GAGGAGGGCAAAGTGGTAAA	97,7%	AF057040.1

### Ethical Note

This study was approved by the Ethics Commission for Animal Use (CEUA) at Universidade de Passo Fundo, UPF, Passo Fundo, RS, Brazil (Protocol 008/2017) and met the guidelines of the National Council for the Control of Animal Experimentation (CONCEA).

### Statistical analysis

Data of time spent in the new object segment exhibited unequal variance between treatment groups and was compared using a Wald-Wolfowits rank test. All other data of behavioral phenotypes were compared by Student’s t test or by Mann-Whitney test depending on the data normality (assessed by the Bartlett’s test). In all experiments, P was set at <0.05.

## Electronic supplementary material

Dataset 1
