# Supplementary material for: First description of behavior and immune system relationship in fish
Source: Sci Rep. 2018 Jan 16;8:846. doi: 10.1038/s41598-018-19276-3 (PMC5770431; doi:10.1038/s41598-018-19276-3)
Supplement: Supplementary file 1 — Dataset 1 [file 41598_2018_19276_MOESM1_ESM.doc]

Supplementary information

**First description of behavior and immune system relationship in fish**

Karina Kirsten, Débora Fior, Luiz Carlos Kreutz, Leonardo José Gil Barcellos

**Table 1. Time (s) spent in the novel object zone - Novel object test**

| HRN | LRN |
| --- | --- |
| 0 | 15 |
| 0 | 5 |
| 0 | 2 |
| 0 | 23 |
| 0 | 14 |
| 0 | 71 |
| 0 | 2 |
| 0 | 46 |
| 0 | 132 |
| 0 | 79 |
| 0 | 84 |
| 0 | 6 |
| 0 | 112 |
| 0 | 26 |
| 0 | 59 |
| 0 | 25 |
| 0 | 30 |
| 0 | 3 |
| 0 | 13 |
| 0 | 38 |
| 0 | 28 |
| 0 | 10 |
| 0 | 95 |
| 0 | 81 |

|  |  |
| --- | --- |
| P value | < 0,0001 |
| P value summary | **** |
| Significantly different? (P < 0.05) | Yes |

|  |  |  |  | Z | Valor de P |  |  |  |  |
| --- | --- | --- | --- | --- | --- | --- | --- | --- | --- |
| 24 | 24 | 41,62500 | 0,00 | -6,71131 | 0,000000 | 6,565411 | 0,000000 | 2 | 0 |

Wald-Wolfowitz Rank Test

**Table 2. Distance Traveled (m) –** **Novel object test**

| HRN | LRN |
| --- | --- |
| 10.660 | 5.586 |
| 8.244 | 16.664 |
| 7.481 | 13.269 |
| 7.210 | 13.844 |
| 11.454 | 17.006 |
| 5.973 | 11.445 |
| 7.860 | 16.053 |
| 11.710 | 20.177 |
| 10.701 | 12.025 |
| 9.100 | 12.324 |
| 5.208 | 16.787 |
| 7.758 | 17.516 |
| 14.137 | 11.686 |
| 7.222 | 19.341 |
| 5.262 | 21.034 |
| 10.857 | 26.015 |
| 10.602 | 26.900 |
| 13.415 | 8.928 |
| 11.692 | 14.677 |
| 9.768 | 14.782 |
| 5.845 | 24.311 |
| 17.254 | 10.561 |
| 13.581 | 19.687 |
| 12.475 | 25.460 |

| Table Analyzed | Distance |
| --- | --- |
|  |  |
| Column A | Neophobic |
| vs. | vs, |
| Column B | NEB |
|  |  |
| Mann Whitney test |  |
| P value | < 0,0001 |
| Exact or approximate P value? | Exact |
| P value summary | **** |
| Significantly different? (P < 0.05) | Yes |
| One- or two-tailed P value? | Two-tailed |
| Sum of ranks in column A,B | 385,0 , 791,0 |
| Mann-Whitney U | 85,00 |
|  |  |
| Difference between medians |  |
| Median of column A | 10,19 |
| Median of column B | 16,36 |
| Difference: Actual | -6,174 |
| Difference: Hodges-Lehmann | -6,255 |
|  |  |

**Table 3. Line crossings - Novel object test**

| HRN | LRN |
| --- | --- |
| 194 | 80 |
| 109 | 242 |
| 82 | 232 |
| 78 | 220 |
| 258 | 251 |
| 67 | 166 |
| 10 | 195 |
| 169 | 251 |
| 127 | 176 |
| 124 | 121 |
| 31 | 238 |
| 67 | 216 |
| 215 | 149 |
| 111 | 298 |
| 100 | 266 |
| 211 | 327 |
| 147 | 402 |
| 265 | 60, |
| 186 | 237 |
| 136 | 194 |
| 132 | 274 |
| 232 | 168 |
| 277 | 347 |
| 264 | 398 |

| Table Analyzed | Line crossing |
| --- | --- |
|  |  |
| Column A | Neophobic |
| vs. | vs, |
| Column B | NEB |
|  |  |
| Unpaired t test |  |
| P value | 0,0015 |
| P value summary | ** |
| Significantly different? (P < 0.05) | Yes |
| One- or two-tailed P value? | Two-tailed |
| t, df | t=3,366 df=46 |
|  |  |
| How big is the difference? |  |
| Mean ± SEM of column A | 149,7 ± 15,76 N=24 |
| Mean ± SEM of column B | 229,5 ± 17,72 N=24 |
| Difference between means | -79,83 ± 23,72 |
| 95% confidence interval | -127,6 to -32,10 |
| R square | 0,1976 |
|  |  |
| F test to compare variances |  |
| F,DFn, Dfd | 1,265, 23, 23 |
| P value | 0,5772 |
| P value summary | ns |
| Significantly different? (P < 0.05) | No |

**Table 4. Rotations - Novel object test**

| HRN | LRN |
| --- | --- |
| 8 | 30 |
| 11 | 30 |
| 26 | 26 |
| 19 | 40 |
| 42 | 58 |
| 9 | 20 |
| 11 | 51 |
| 35 | 63 |
| 29 | 34 |
| 25 | 28 |
| 17 | 56 |
| 31 | 39 |
| 32 | 49 |
| 17 | 50 |
| 23 | 58 |
| 49 | 65 |
| 17 | 65 |
| 38 | 28 |
| 30 | 28 |
| 32 | 15 |
| 4 | 44 |
| 29 | 43 |
| 56 | 36 |
| 32 | 67 |

| Table Analyzed | Rotations |
| --- | --- |
|  |  |
| Column A | Neophobic |
| vs. | vs, |
| Column B | NEB |
|  |  |
| Unpaired t test |  |
| P value | 0,0002 |
| P value summary | *** |
| Significantly different? (P < 0.05) | Yes |
| One- or two-tailed P value? | Two-tailed |
| t, df | t=4,048 df=46 |
|  |  |
| How big is the difference? |  |
| Mean ± SEM of column A | 25,92 ± 2,667 N=24 |
| Mean ± SEM of column B | 42,63 ± 3,151 N=24 |
| Difference between means | -16,71 ± 4,128 |
| 95% confidence interval | -25,02 to -8,399 |
| R square | 0,2626 |
|  |  |
| F test to compare variances |  |
| F,DFn, Dfd | 1,396, 23, 23 |
| P value | 0,4300 |
| P value summary | ns |
| Significantly different? (P < 0.05) | No |

**Table 5. Time (s) spent at bottom – Novel object test**

| HRN | LRN |
| --- | --- |
| 132 | 354 |
| 42 | 157 |
| 253 | 227 |
| 308 | 284 |
| 347 | 237, |
| 0 | 321 |
| 0 | 187 |
| 87 | 111 |
| 346 | 254 |
| 64 | 333 |
| 4 | 251 |
| 16 | 142 |
| 251 | 341 |
| 52 | 324 |
| 125 | 141 |
| 58 | 102 |
| 97 | 222 |
| 290 | 54 |
| 144 | 198 |
| 192 | 213, |
| 361 | 152 |
| 111 | 177 |
| 87 | 268 |
| 218 | 348 |

| Table Analyzed | TEMPO FUNDO |
| --- | --- |
|  |  |
| Column B | NEB |
| vs. | vs, |
| Column A | Neophobic |
|  |  |
| Mann Whitney test |  |
| P value | 0,0160 |
| Exact or approximate P value? | Exact |
| P value summary | * |
| Significantly different? (P < 0.05) | Yes |
| One- or two-tailed P value? | Two-tailed |
| Sum of ranks in column A,B | 472,0 , 704,0 |
| Mann-Whitney U | 172,0 |
|  |  |
| Difference between medians |  |
| Median of column A | 118,0 |
| Median of column B | 224,5 |
| Difference: Actual | 106,5 |
| Difference: Hodges-Lehmann | 89,50 |

**Table 6. Relative mRNA gene expression IL-1β – Novel object test**

| HRN | LRN |
| --- | --- |
| 5.414000 | 3.660000 |
| 3.181000 | 0.642000 |
| 7.072000 | 2.935000 |
| 5.997000 | 3.626000 |
| 5.592000 | 1.654000 |
| 3.984000 | 5.595000 |

| Table Analyzed | IL-1B |
| --- | --- |
|  |  |
| Column A | Neophobic |
| vs. | vs, |
| Column B | NEB |
|  |  |
| Unpaired t test |  |
| P value | 0,0370 |
| P value summary | * |
| Significantly different? (P < 0.05) | Yes |
| One- or two-tailed P value? | Two-tailed |
| t, df | t=2,405 df=10 |
|  |  |
| How big is the difference? |  |
| Mean ± SEM of column A | 5,207 ± 0,5743 N=6 |
| Mean ± SEM of column B | 3,019 ± 0,7056 N=6 |
| Difference between means | 2,188 ± 0,9097 |
| 95% confidence interval | 0,1610 to 4,215 |
| R square | 0,3665 |
|  |  |
| F test to compare variances |  |
| F,DFn, Dfd | 1,510, 5, 5 |
| P value | 0,6623 |
| P value summary | ns |
| Significantly different? (P < 0.05) | No |

**Table 7.** Relative mRNA gene expression IL-10 – Novel object test

| HRN | LRN |
| --- | --- |
| T | 13.340240 |
| 14.198950 | 11.533130 |
| 8.740480 | 87.895190 |
| 9.303110 | 12.190730 |
| 5.806680 | 12.190730 |

| Table Analyzed | IL10 |
| --- | --- |
|  |  |
| Column A | Neophobic |
| vs. | vs, |
| Column B | NEB |
|  |  |
| Mann Whitney test |  |
| P value | 0,0437 |
| Exact or approximate P value? | Exact |
| P value summary | * |
| Significantly different? (P < 0.05) | Yes |
| One- or two-tailed P value? | One-tailed |
| Sum of ranks in column A,B | 19,00 , 36,00 |
| Mann-Whitney U | 4,000 |
|  |  |
| Difference between medians |  |
| Median of column A | 8,740 |
| Median of column B | 12,19 |
| Difference: Actual | -3,450 |
| Difference: Hodges-Lehmann | -5,726 |

**Table 8. Relative mRNA gene expression TNF-α** – Novel object test

| HRN | LRN |
| --- | --- |
| 5.013000 | 2.137000 |
| 4.044000 | 2.801000 |
| 2.355000 | 6.302000 |
| 4.100000 | 3.151000 |
| 6.130000 | 1.353000 |
| 0.667000 | 2.050000 |

| Table Analyzed | TNF |
| --- | --- |
|  |  |
| Column A | Neophobic |
| vs. | vs, |
| Column B | NEB |
|  |  |
| Unpaired t test |  |
| P value | 0,4972 |
| P value summary | ns |
| Significantly different? (P < 0.05) | No |
| One- or two-tailed P value? | Two-tailed |
| t, df | t=0,7045 df=10 |
|  |  |
| How big is the difference? |  |
| Mean ± SEM of column A | 3,718 ± 0,7940 N=6 |
| Mean ± SEM of column B | 2,966 ± 0,7146 N=6 |
| Difference between means | 0,7525 ± 1,068 |
| 95% confidence interval | -1,628 to 3,133 |
| R square | 0,04728 |
|  |  |
| F test to compare variances |  |
| F,DFn, Dfd | 1,235, 5, 5 |
| P value | 0,8227 |
| P value summary | ns |
| Significantly different? (P < 0.05) | No |

**Table 9.** Relative mRNA gene expression IL-4 – Novel object test

| HRN | LRN |
| --- | --- |
| 23.519000 | 7.867000 |
| 5.048000 | 16.402000 |
| 6.130000 | 15.303000 |
| 3.988000 | 8.490000 |
| 10.027000 | 7.867000 |
| 6.479000 | 4.245000 |

| Table Analyzed | IL4 |
| --- | --- |
|  |  |
| Column A | Neophobic |
| vs. | vs, |
| Column B | NEB |
|  |  |
| Unpaired t test |  |
| P value | 0,8203 |
| P value summary | ns |
| Significantly different? (P < 0.05) | No |
| One- or two-tailed P value? | Two-tailed |
| t, df | t=0,2332 df=10 |
|  |  |
| How big is the difference? |  |
| Mean ± SEM of column A | 9,199 ± 2,983 N=6 |
| Mean ± SEM of column B | 10,03 ± 1,946 N=6 |
| Difference between means | -0,8305 ± 3,562 |
| 95% confidence interval | -8,766 to 7,105 |
| R square | 0,005408 |
|  |  |
| F test to compare variances |  |
| F,DFn, Dfd | 2,350, 5, 5 |
| P value | 0,3701 |
| P value summary | ns |
| Significantly different? (P < 0.05) | No |

**Table 10.** Relative mRNA gene expression IL-12 – Novel object test

| HRN | LRN |
| --- | --- |
| 9.741000 | 23.774000 |
| 16.729000 | 6.565000 |
| 19.949000 | 4.879000 |
| 15.106000 | 17.245000 |
| 2.458000 | 22.350000 |

| Table Analyzed | IL-12 |
| --- | --- |
|  |  |
| Column A | Neophobic |
| vs. | vs, |
| Column B | NEB |
|  |  |
| Unpaired t test |  |
| P value | 0,6756 |
| P value summary | ns |
| Significantly different? (P < 0.05) | No |
| One- or two-tailed P value? | Two-tailed |
| t, df | t=0,4342 df=8 |
|  |  |
| How big is the difference? |  |
| Mean ± SEM of column A | 12,80 ± 3,067 N=5 |
| Mean ± SEM of column B | 14,96 ± 3,935 N=5 |
| Difference between means | -2,166 ± 4,989 |
| 95% confidence interval | -13,67 to 9,339 |
| R square | 0,02302 |
|  |  |
| F test to compare variances |  |
| F,DFn, Dfd | 1,645, 4, 4 |
| P value | 0,6413 |
| P value summary | ns |
| Significantly different? (P < 0.05) | No |

**Table 11. Relative mRNA gene expression INF-γ** – Novel object test

| HRN | LRN |
| --- | --- |
| 5.418000 | 6.295000 |
| 1.727000 | 6.340000 |
| 7.977000 | 2.978000 |
| 3.326000 | 3.671000 |
| 18.337000 | 1.672000 |
| 3.461000 | 1.916000 |

| Table Analyzed | INF |
| --- | --- |
|  |  |
| Column A | Neophobic |
| vs. | vs, |
| Column B | NEB |
|  |  |
| Mann Whitney test |  |
| P value | 0,4740 |
| Exact or approximate P value? | Exact |
| P value summary | ns |
| Significantly different? (P < 0.05) | No |
| One- or two-tailed P value? | Two-tailed |
| Sum of ranks in column A,B | 44,00 , 34,00 |
| Mann-Whitney U | 13,00 |
|  |  |
| Difference between medians |  |
| Median of column A | 4,440 |
| Median of column B | 3,325 |
| Difference: Actual | 1,115 |
| Difference: Hodges-Lehmann | 1,591 |

**Table 12. Time (s) spent in the coenspecific segment – Social preference test**

| Preference | No preference |
| --- | --- |
| 43.1 | 31.4 |
| 49.8 | 37.0 |
| 60.0 | 34.0 |
| 43.6 | 32.8 |
| 57.4 | 7.9 |
| 56.5 | 30.5 |
| 44.6 | 29.8 |
| 60.0 | 38.6 |
| 55.1 | 25.6 |
| 59.0 | 4.6 |
| 56.5 | 11.0 |
| 51.9 | 0.1 |
| 45.4 | 24.7 |
| 57.7 | 20.4 |
| 60.0 | 38.5 |
| 49.5 | 33.1 |
| 60.0 | 28.8 |
| 54.7 | 35.6 |
| 44.8 | 7.4 |
| 51.7 | 7.3 |
| 56.7 | 8.5 |
| 58.0 | 25.6 |
| 48.4 | 36.9 |
| 51.0 | 25.1 |

| Table Analyzed | Unpaired t test data |
| --- | --- |
|  |  |
| Column B | No preference |
| vs. | vs, |
| Column A | Preference |
|  |  |
| Mann Whitney test |  |
| P value | < 0,0001 |
| Exact or approximate P value? | Exact |
| P value summary | **** |
| Significantly different? (P < 0.05) | Yes |
| One- or two-tailed P value? | Two-tailed |
| Sum of ranks in column A,B | 876,0 , 300,0 |
| Mann-Whitney U | 0,0 |
|  |  |
| Difference between medians |  |
| Median of column A | 54,90 |
| Median of column B | 27,20 |
| Difference: Actual | -27,70 |
| Difference: Hodges-Lehmann | -26,30 |

**Table 13. Relative mRNA gene expression IL-1β** – Social preference test

| Preference | No preference |
| --- | --- |
| 6.440000 | 5.570000 |
| 5.850000 | 10.400000 |
| 2.860000 | 4.920000 |
| 2.230000 | 6.910000 |
| 5.090000 | 2.530000 |
| 6.180000 | 8.330000 |

| Table Analyzed | IS-IL1 |
| --- | --- |
|  |  |
| Column A | Preference |
| vs. | vs, |
| Column B | No preference |
|  |  |
| Unpaired t test |  |
| P value | 0,2419 |
| P value summary | ns |
| Significantly different? (P < 0.05) | No |
| One- or two-tailed P value? | Two-tailed |
| t, df | t=1,244 df=10 |
|  |  |
| How big is the difference? |  |
| Mean ± SEM of column A | 4,775 ± 0,7336 N=6 |
| Mean ± SEM of column B | 6,443 ± 1,123 N=6 |
| Difference between means | -1,668 ± 1,341 |
| 95% confidence interval | -4,657 to 1,320 |
| R square | 0,1340 |
|  |  |
| F test to compare variances |  |
| F,DFn, Dfd | 2,343, 5, 5 |
| P value | 0,3716 |
| P value summary | ns |
| Significantly different? (P < 0.05) | No |

**Table 14.** Relative mRNA gene expression IL-10 – Social preference test

| Preference | No preference |
| --- | --- |
| 6.200000 | 10.600000 |
| 5.100000 | 5.500000 |
| 1.300000 | 1.900000 |
| 0.400000 | 10.100000 |
| 6.400000 | 1.300000 |
| 5.400000 | 4.100000 |

| Table Analyzed | IL-10 |
| --- | --- |
|  |  |
| Column A | Preference |
| vs. | vs, |
| Column B | No preference |
|  |  |
| Unpaired t test |  |
| P value | 0,4733 |
| P value summary | ns |
| Significantly different? (P < 0.05) | No |
| One- or two-tailed P value? | Two-tailed |
| t, df | t=0,7451 df=10 |
|  |  |
| How big is the difference? |  |
| Mean ± SEM of column A | 4,133 ± 1,063 N=6 |
| Mean ± SEM of column B | 5,583 ± 1,630 N=6 |
| Difference between means | -1,450 ± 1,946 |
| 95% confidence interval | -5,786 to 2,886 |
| R square | 0,05260 |
|  |  |
| F test to compare variances |  |
| F,DFn, Dfd | 2,350, 5, 5 |
| P value | 0,3701 |
| P value summary | ns |
| Significantly different? (P < 0.05) | No |

**Table 15. Relative mRNA gene expression TNF-α** – Social preference test

| Preference | No preference |
| --- | --- |
| 6.760000 | 2.600000 |
| 1.450000 | 17.720000 |
| 0.920000 | 1.160000 |
| 3.270000 | 12.110000 |
| 5.690000 | 2.420000 |
| 2.110000 | 3.780000 |

| Table Analyzed | TNF-alfa |
| --- | --- |
|  |  |
| Column A | Preference |
| vs. | vs, |
| Column B | No preference |
|  |  |
| Mann Whitney test |  |
| P value | 0,4740 |
| Exact or approximate P value? | Exact |
| P value summary | ns |
| Significantly different? (P < 0.05) | No |
| One- or two-tailed P value? | Two-tailed |
| Sum of ranks in column A,B | 34,00 , 44,00 |
| Mann-Whitney U | 13,00 |
|  |  |
| Difference between medians |  |
| Median of column A | 2,690 |
| Median of column B | 3,190 |
| Difference: Actual | -0,5000 |
| Difference: Hodges-Lehmann | -1,060 |

**Table 16.** Relative mRNA gene expression IL-4 – Social preference test

| Preference | No preference |
| --- | --- |
| 1.850000 | 1.660000 |
| 0.780000 | 1.560000 |
| 0.360000 | 0.790000 |
| 0.570000 | 1.610000 |
| 0.420000 | 0.890000 |
| 1.770000 | 0.910000 |

| Table Analyzed | IL4 |
| --- | --- |
|  |  |
| Column A | Preference |
| vs. | vs, |
| Column B | No preference |
|  |  |
| Unpaired t test |  |
| P value | 0,4093 |
| P value summary | ns |
| Significantly different? (P < 0.05) | No |
| One- or two-tailed P value? | Two-tailed |
| t, df | t=0,8612 df=10 |
|  |  |
| How big is the difference? |  |
| Mean ± SEM of column A | 0,9583 ± 0,2759 N=6 |
| Mean ± SEM of column B | 1,237 ± 0,1683 N=6 |
| Difference between means | -0,2783 ± 0,3232 |
| 95% confidence interval | -0,9985 to 0,4418 |
| R square | 0,06904 |
|  |  |
| F test to compare variances |  |
| F,DFn, Dfd | 2,689, 5, 5 |
| P value | 0,3016 |
| P value summary | ns |
| Significantly different? (P < 0.05) | No |

**Table 17.** Relative mRNA gene expression IL-12 – Social preference test

| Preference | No preference |
| --- | --- |
| 2.899000 | 1.501000 |
| 3.173000 | 2.524000 |
| 0.943000 | 0.905000 |
| 2.388000 | 2.472000 |
| 3.933000 | 1.334000 |
| 2.577000 | 2.559000 |

| Table Analyzed | IL-12 |
| --- | --- |
|  |  |
| Column A | Preference |
| vs. | vs, |
| Column B | No preference |
|  |  |
| Unpaired t test |  |
| P value | 0,0785 |
| P value summary | ns |
| Significantly different? (P < 0.05) | No |
| One- or two-tailed P value? | One-tailed |
| t, df | t=1,530 df=10 |
|  |  |
| How big is the difference? |  |
| Mean ± SEM of column A | 2,652 ± 0,4070 N=6 |
| Mean ± SEM of column B | 1,883 ± 0,2954 N=6 |
| Difference between means | 0,7697 ± 0,5029 |
| 95% confidence interval | -0,3509 to 1,890 |
| R square | 0,1898 |
|  |  |
| F test to compare variances |  |
| F,DFn, Dfd | 1,898, 5, 5 |
| P value | 0,4989 |
| P value summary | ns |
| Significantly different? (P < 0.05) | No |

**Table 18. Relative mRNA gene expression INF-γ** – Social preference test

| Preference | No preference |
| --- | --- |
| 8.800000 | 2.300000 |
| 9.800000 | 2.100000 |
| 3.100000 | 3.600000 |
| 7.600000 | 5.100000 |
| 8.500000 | 2.700000 |
| 8.700000 | 4.000000 |

| Table Analyzed | INF |
| --- | --- |
|  |  |
| Column A | Preference |
| vs. | vs, |
| Column B | No preference |
|  |  |
| Unpaired t test |  |
| P value | 0,0021 |
| P value summary | ** |
| Significantly different? (P < 0.05) | Yes |
| One- or two-tailed P value? | Two-tailed |
| t, df | t=4,118 df=10 |
|  |  |
| How big is the difference? |  |
| Mean ± SEM of column A | 7,750 ± 0,9732 N=6 |
| Mean ± SEM of column B | 3,300 ± 0,4698 N=6 |
| Difference between means | 4,450 ± 1,081 |
| 95% confidence interval | 2,042 to 6,858 |
| R square | 0,6290 |
|  |  |
| F test to compare variances |  |
| F,DFn, Dfd | 4,292, 5, 5 |
| P value | 0,1358 |
| P value summary | ns |
| Significantly different? (P < 0.05) | No |
